# Strategy for scheduled downtime of hospital information system utilizing third-party applications

**DOI:** 10.1186/s12911-024-02710-0

**Published:** 2024-10-11

**Authors:** Inhae Jo, Woojin Kim, Younghee Lim, Eunjeong Kang, Jinung Kim, Hyekyung Chung, Jihae Kim, Eunhye Kang, Yoon Bin Jung

**Affiliations:** 1 Center for Digital Health Strategy, Division of Digital Health, Yonsei University Health System, Seoul, Korea; 2 Center for Information Services, Division of Digital Health, Yonsei University Health System, Seoul, Korea; 3 Department of Surgery, Yonsei University College of Medicine, 50-1 Yonsei-ro, Seodaemun-gu, Seoul, 03722 Korea; 4 Institue for Innovation in Digital Healthcare, Yonsei University, Seoul, Korea

**Keywords:** Hospital information system, Electronic medical records, Order communication system

## Abstract

**Background:**

The widespread adoption of Hospital Information Systems (HIS) has brought significant benefits in healthcare quality and workflow efficiency. However, downtimes for system maintenance are inevitable and pose a considerable challenge to continuous patient care. Existing strategies, including manual prescription methods, are no longer effective due to increasing reliance on digital systems.

**Method:**

This study implemented two main strategies to mitigate the impact of scheduled downtimes. First, we created an “Emergency query program” that switches to a read-only backup server during downtimes, allowing clinicians to view essential patient data. Second, an “Emergency prescription system” was developed based on the Microsoft Power Platform and integrated into Microsoft Teams. This allows clinicians to perform digital prescriptions even during downtimes.

**Results:**

During a planned 90-minute downtime, 282 users accessed the Emergency Prescription System, resulting in 22 prescriptions from various departments. Average times for prescription confirmation and completion were 8 min and 3 s, and 18 min and 40 s, respectively. A post-downtime evaluation revealed high user satisfaction.

**Conclusion:**

Essential maintenance-induced HIS downtimes are inherently disruptive to patient care process. Our deployment of an emergency query program and a Microsoft Teams-integrated emergency prescription system demonstrated robust care continuity during HIS downtime.

## Background

Since the advent of Electronic Medical Records (EMR) systems in the 1960s, many healthcare institutions have transitioned from the previous method of managing patient records in paper form to utilizing core information technologies to enhance medical staff convenience, improve the quality of care, and establish digital hospitals [1]. The large-scale adoption of Electronic Medical Records in the United States has been significantly influenced by the Health Information Technology for Economic and Clinical Health (HITECH) Act, which was enacted in 2009 as part of the American Recovery and Reinvestment Act. The HITECH Act aimed to promote the adoption and meaningful use of health information technology, particularly EMRs, by providing financial incentives to healthcare providers [2]. HITECH Act and EMR adoption has led to a dramatic increase in the number of healthcare facilities implementing these systems, resulting in improved patient care and streamlined operations across the industry. Based on various studies indicating that the introduction of EMRs improves the quality of care, patient outcomes, and healthcare productivity [3, 4], approximately 88% of hospitals in the United States adopted EMRs as a fundamental system for health care informatization [5]. 

Electronic Medical Records systems are integral to modern healthcare, but downtime or outages are an inevitable occurrence in hospitals for several reasons. EMR systems are highly complex and involve numerous interconnected components, including hardware, software, and network infrastructure. This complexity increases the likelihood of failures due to software bugs, compatibility issues, or hardware malfunctions [6]. And regular maintenance and software updates are essential for optimal performance and security. However, these processes can lead to temporary outages. Scheduling these updates can be challenging, especially in busy hospital environments where continuous access to patient records is critical [7]. Given the continuous nature of patient care in healthcare facilities, Hospital Information System(HIS) outage can affect the quality of care and patient safety. Larsen et al. reported that approximately 46% of medical incidents occurring during EMR downtime were not appropriately managed [8]. 

To respond to such HIS downtime, big commercial EMR vendors provide failover systems. Despite the availability of failover systems offered by large commercial EMR vendors, many hospitals face challenges in utilizing these solutions. These challenges often stem from inadequate training, integration issues with existing workflows, and the high costs associated with implementing and maintaining these systems. Implementing robust failover systems can be prohibitively expensive, particularly for smaller hospitals or those with limited budgets. The costs associated with additional hardware, software licensing, and ongoing maintenance can strain financial resources. And the integration of failover systems often requires specialized knowledge and expertise. Many hospitals may lack the necessary IT staff or resources to implement and maintain these complex systems effectively. Moreover, transitioning to a failover system necessitates training staff and managing change within the organization. This process can be time-consuming and may disrupt normal operations, leading to resistance from employees. Especially for hospitals that use homegrown solutions, it is very difficult to establish their own failover systems, so it is very important to easily develop and operate solutions that can respond to HIS downtime.

Especially for hospitals that implement homegrown solutions often focus on optimizing paper-based processes during downtime to maintain continuity of care. For instance, staff may rely on printed forms and manual documentation to track patient information, medication administration, and other essential data until the EMR system is restored [9]. However, the limitations of paper-based processes—such as the potential for errors, difficulty in data retrieval, and challenges in maintaining comprehensive records—underscore the necessity for hospitals to develop and operate in-house solutions tailored to their specific needs. These custom solutions can enhance the reliability of information collection and facilitate smoother transitions back to electronic systems post-downtime [10]. By investing in such tailored approaches, hospitals can better prepare for HIS outages, ensuring that patient care remains uninterrupted and that staff can efficiently navigate the complexities of healthcare delivery in a hybrid environment. Our institution planned for an HIS server upgrade and database enhancement, which required scheduled HIS downtime. During this period, we aimed to maintain continuity of care and effectively replace the traditional manual prescription processes by developing an emergency query program and an emergency prescription system utilizing third party software to respond efficiently to HIS downtime.

## Methods

### Study setting

This study was conducted in response to the HIS downtime that occurred due to the hospital information system infrastructure upgrade at Severance Hospital, Seoul. Severance Hospital constitutes a tertiary care facility, comprising 2,462 beds and employing a workforce of 1,329 physicians who operate within 25 specialized medical departments. This institution represents a prominent medical establishment in Korea, registering an annual outpatient volume of 2.6 million and accommodating 760,000 inpatient admissions. Severance Hospital implemented homegrown EMR solution, termed ‘u-Severance,’ in the year 2005, which operates on the Microsoft Windows Server platform and utilizes an MS-SQL relational database(DB). The architecture of this system is predicated on .NET framework and was developed employing MS Visual Studio .NET with C# functioning as the integrated development environment. Subsequent to its inaugural deployment in 2005, the system underwent an enhancement to u-Severance 2.0 in 2012, and further upgraded to the current version, u-Severance 3.0, in 2015, with major UI improvements.

### Scheduled downtime

Our hospital undertook the ‘HIS DB Enhancement Project’ to upgrade the infrastructure of the HIS. The scope of the project included replacing outdated servers with new ones, upgrading the Microsoft SQL Server version, integrating the databases of the EMR and Order Communication System(OCS), and configuring Always On Availability Groups for a high-availability environment in SQL server. The project required the shutdown of the HIS operational servers, making HIS downtime inevitable. To minimize the impact of the HIS downtime on clinical operations, it was decided to carry out the project during a period with minimal clinical activity. The hospital leadership determined that Sunday, when no outpatient services are running, would be the most optimal day for the work. Additionally, they decided to initiate the HIS downtime and begin the work at 2 AM, a time when the number of surgeries and emergency room patients is at its lowest. For these reasons, the project was scheduled to begin at 2 AM on August 13, 2023, with the expected duration estimated to be a minimum of 90 min and a maximum of 150 min.

### Emergency query program

**Fig. 1:**
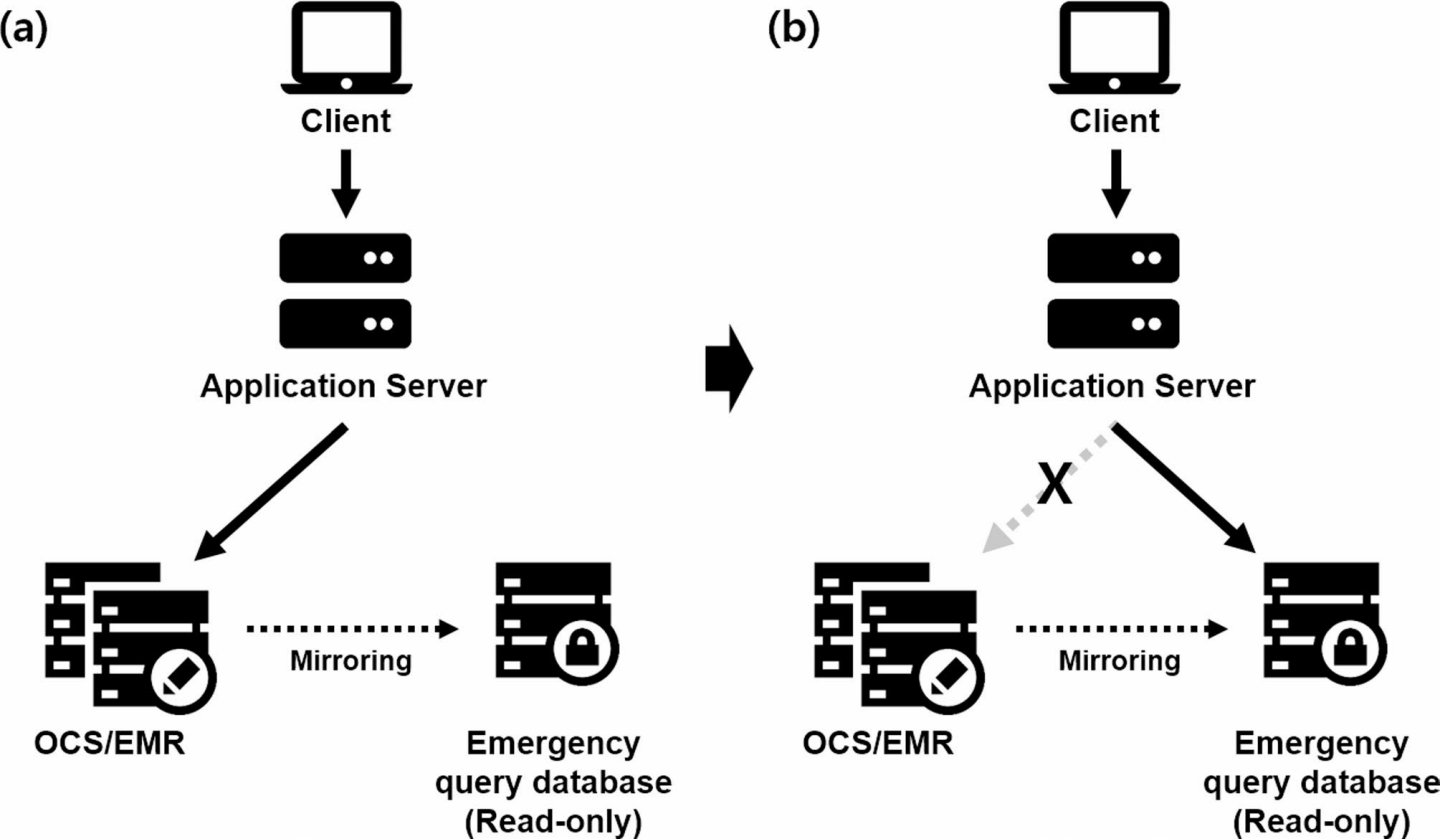
Emergency query program operational structure diagram. (**a**) Regular operating environment, (**b**) HIS downtime environment

Our institution has implemented database duplication for the EMR and computerized order entry system to ensure stable storage and operation of data, regularly mirroring data from the operational server to the backup server for continuity. Prior to a scheduled HIS downtime, the application server, which connects the clients and the EMR and prescription delivery system’s operational server, is switched to the backup server. Certain interfaces have been configured to enable access to patient information and previous medical records during the downtime. However, to prevent discrepancies between the operational and backup server data due to changes during the outage, the backup server’s database operates in read-only mode (Fig. 1).

### Microsoft teams-integrated emergency prescription system

In the event of computational failure, where the HIS is inaccessible, our institution has maintained patient care by operating a manual prescription process where prescriptions are written on paper and physically delivered to the clinical support departments. Despite this, difficulties such as loss of prescription information and interdepartmental communication challenges necessitated a computerized prescription and execution process usable during HIS downtime. To address this need, we developed new software using a cloud-based 3rd party application independent of the operational server status, similar to the existing HIS. Specifically, we utilized the Microsoft Power Platform, which includes Power Apps and Power Automate, as essential tools for developing our solution. Power Apps enables users to create low-code custom applications that can streamline processes and improve productivity, while Power Automate facilitates the automation of workflows across various applications and services. This integration not only enhances efficiency but also allows for seamless data management and communication among different platforms. Subsequently, the new solution was deployed in an in-app format within the Microsoft Teams Solution, which is used as an internal messenger in the hospital, allowing it to be operated on a separate platform even during HIS downtime. By leveraging these third-party tools, we were able to build the necessary solution without extensive programming knowledge. While other platforms from vendors such as Salesforce, Mendix, and Appian exist, we determined that leveraging Microsoft’s existing licenses already held by the hospital would be more cost-effective.

**Fig. 2:**
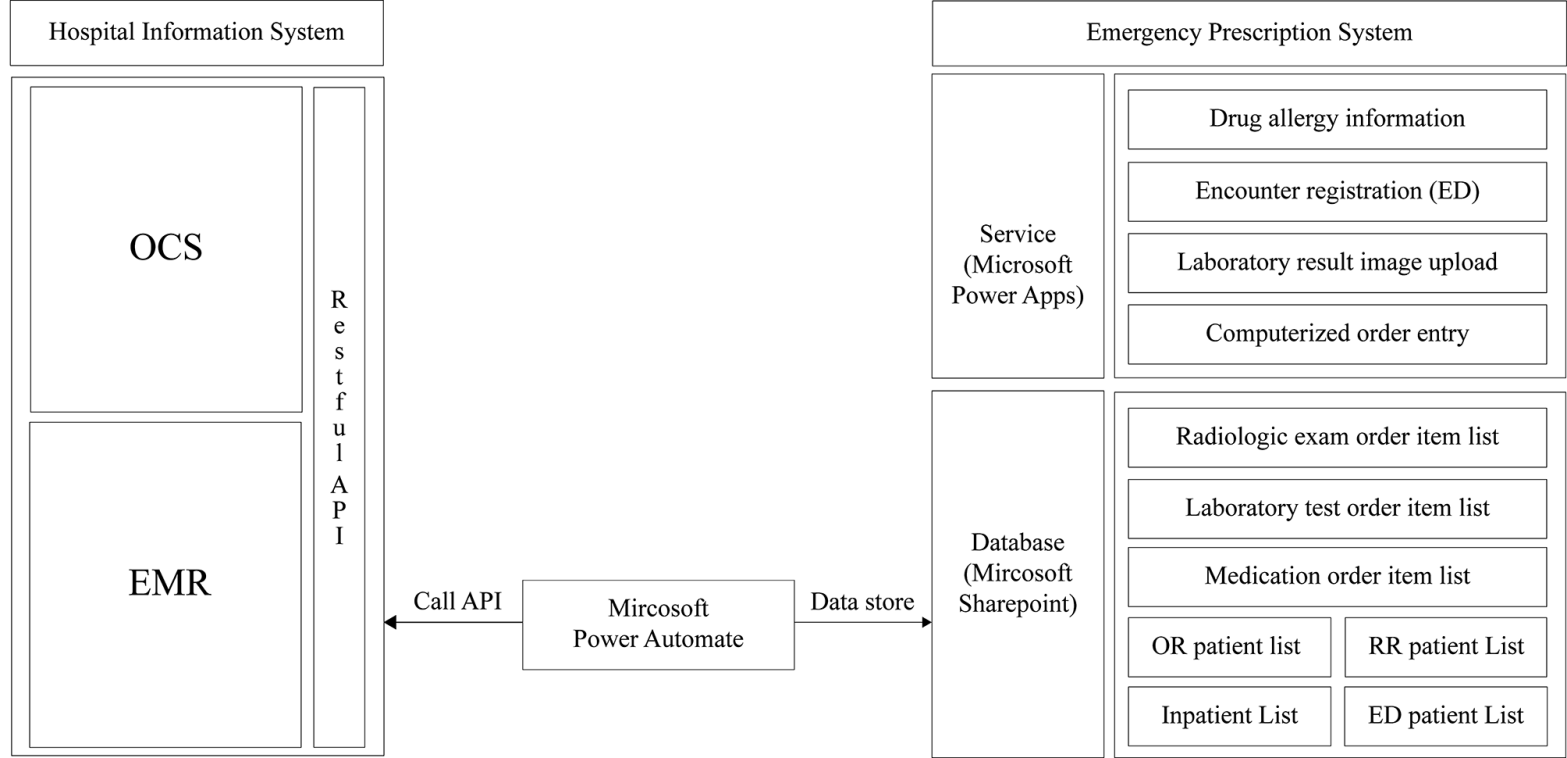
Data integration architecture between HIS and emergency prescription system

**Fig. 3:**
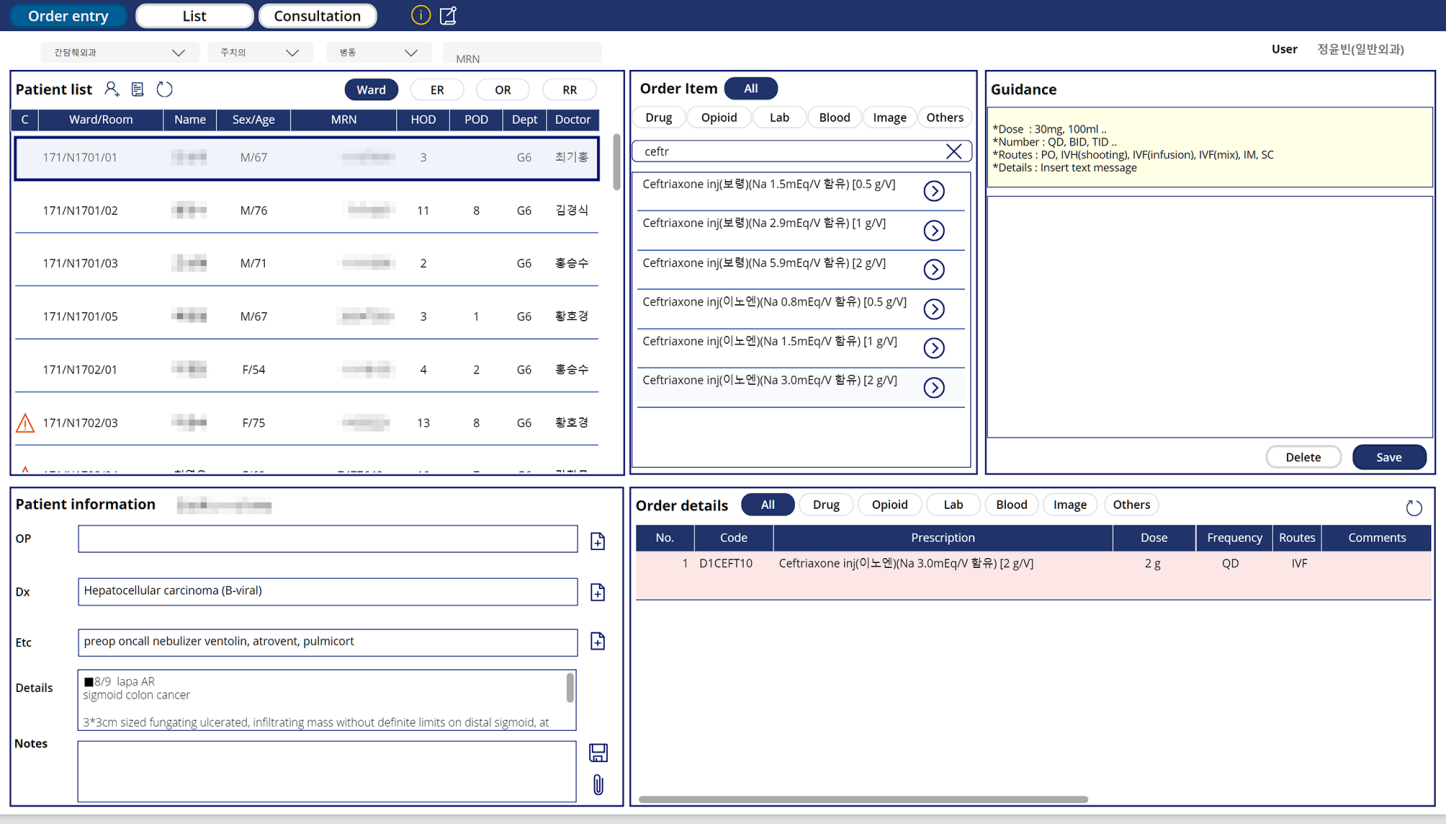
Emergency prescription system interface layout on Microsoft Power Apps

We ensured user convenience by replicating similar screen configurations and functionalities of the existing HIS. Patients were categorized into inpatient, emergency room, operational room, and recovery room, with the ability to query based on department, primary physician, ward, and medical record number. In order to replace the HIS during downtime, the new solution needed to perform functions similar to those of the HIS. To achieve this, it was necessary to synchronize the patient list, as well as the list of medications and tests available for prescription, with the new solution. We developed RESTful APIs to query the patient list and prescription data from the HIS, and used Power Automate to periodically call these APIs and store the data in Microsoft SharePoint, which serves as the database for the emergency prescription system. However, since this project involved a planned downtime, the APIs were manually called just before the start of the work to update the database with the most recent patient information (Fig. 2). Prescriptions were categorized into medication, laboratory test, radiologic examination, blood product and other examination, structured to minimize errors during HIS downtime by aligning with the standard information in OCS (Fig. 3).

Prescription information entered into the emergency prescription system was digitally transmitted to each clinical support department, where the execution status of each prescription was input into the system to support effective interdepartmental communication (Table 1).

**Table 1:** Prescription Verification and fulfillment process in emergency prescription system

	Prescription Verification and Fulfillment Process					
Order type	Step 1	Step 2	Step 3	Step 4	Step 5	Step 6
Medication	Prescription (Physician)	Prescription confirmation (Nurse)	Prescription receipt (Pharmacy)	Prescription printing (Pharmacy)	Medication dispensing completed (Pharmacy)	Medication dispense request (Nurse)
Laboratory	Prescription (Physician)	Prescription confirmation (Nurse)	Specimen collection and transportation (Nurse)	Specimen reception confirmation (Laboratory)	Results reporting (Laboratory)	Results review (Physician)
Blood products	Prescription (Physician)	Prescription confirmation (Nurse)	Prescription receipt (Blood bank)	Release preparation completion (Blood bank)	Blood product release request (Nurse)	Blood product reception (Nurse)
Radiologic examination (General)	Prescription (Physician)	Prescription confirmation (Nurse)	Consent form acquisition and upload (Nurse)	Patient transport call (Nurse)	Test conducted (Radiology)	Test completion confirmation (Radiology)
Radiologic examination (Portable)	Prescription (Physician)	Prescription confirmation (Nurse)	Consent form Acquisition and upload (Nurse)	Prescription printing (Radiology)	Test conducted (Radiology)	Test completion confirmation (Radiology)
Others	Prescription (Physician)	Prescription confirmation (Nurse)	Patient transport call (Nurse)	Test conducted (Relevant department)	Test completion Confirmation (Relevant department)	

Especially during HIS downtime, for the reporting of laboratory test results, the emergency prescription system offered an alternative to manually writing results on paper in the lab and delivering them to the prescribing department. It allowed results to be scanned and sent as file attachments, facilitating immediate verification remotely and reducing concerns of lost results. After the HIS downtime, it was essential to re-enter the manually prescribed items into the HIS, record whether the prescriptions were executed, and integrate the test results. The manual prescription process with paper prescriptions had a high risk of prescription information loss when re-entering data into the HIS. Therefore, the emergency prescription system digitized prescription records and used an automated process with Microsoft Power Automate to send prescription details to each prescriber’s email, assisting with seamless re-entry into the HIS.

### User training for HIS downtime response process

In preparation for HIS downtime, having a well-crafted plan in place is critically important. It’s essential that all members are informed about the plan and receive training to ensure a successful response [11, 12]. To facilitate the transition from the existing manual prescription process to the emergency prescription system for HIS downtime, our institution prepared and distributed user guides for the program in advance. Additionally, specialized training sessions for doctors, nurses, pharmacists, and other medical personnel were held to familiarize them with the new system before deployment. All relevant departments also engaged in mock drills, simulating prescribing and prescription fulfillment to be fully prepared for the scheduled HIS downtime.

### Post-downtime user Survery

After the HIS downtime ended, an online survey for user feedback on the emergency query program and emergency prescription system was conducted for about two weeks. The online survey was carried out via email targeting the 282 userswithin the hospital who had used the new solution during the HIS downtime, and the staff who received the email participated voluntarily and anonymously in the survey. It was impossible to retrospectively obtain consent from anonymous respondents at the time of this study, and the acquisition of informed consent was waived as all the items specified in 45 CFR (Code of Federal Regulation) 46.116(f) [3] were met. Including the above, this study was approved by the Yonsei University Health System, Severance Hospital, Institutional Review Board.(IRB No. 4-2023-1294).

## Results

Our institution scheduled approximately 90 min of HIS downtime from 2:00 AM to 3:30 AM for the purpose of upgrading the operational server infrastructure and enhancing the database of the HIS. The enhancement involved establishing a high-availability database environment, optimizing database indexes, partitioning and archiving tasks, as well as query optimization efforts. The downtime schedule was communicated in advance to allow for effective departmental preparation. Prior to the downtime, the hospital had 1,936 inpatients, 32 emergency room patients, and 4 emergency surgeries in progress. During the downtime, the emergency query program was accessed by 260 individuals, while the emergency prescription system was used by 282 users. A total of 22 prescriptions were issued: 11 by floor units, 3 by the intensive care unit, 7 by the emergency room, and 1 by the operating room, comprising 10 medication orders, 9 laboratory tests, and 3 for other examinations. The average time for nurse confirmation of a prescription was 8 min and 3 s, and the average time from nurse confirmation to departmental verification was 7 min and 17 s. The completion of prescription execution after issuance took an average of 18 min and 40 s. (Table 2)

**Table 2:** Results of prescriptions executed using the emergency prescription system during HIS downtime

Item	*n*
Total number of prescriptions	22
Prescriptions by department	
Floor unit	11
Intensive care unit	3
Emergency room	7
Operation room	1
Prescriptions by type	
Medication	10
Laboratory	9
Other examination	3
Average prescription execution time	
From doctor’s prescription to nurse’s verification of prescription	8 m 3s
From nurse’s verification of prescription to relevant department’s completion of prescription execution	7 m 17s
From doctor’s prescription to relevant department’s completion of prescription execution	18 m 40s

Post-downtime, user evaluations were collected for the emergency query program and the Microsoft Teams-integrated emergency prescription system. (Table 3) Out of the 282 users invited to participate in the survey, 93 responded to assess the system after the HIS outage. Of these participants, 43 had experienced HIS downtime before, whereas 50 had not. Of the respondents, 34 had experience with manual prescription processes, and 59 did not. 26 indicated that their department’s manual prescription preparations for HIS downtime were inadequate. In terms of executing manual prescriptions during downtime, 11 users believed they could manage without reference materials, 74 felt they could proceed with guidelines, and 8 found it impossible. The feedback suggests that the emergency query program and emergency prescription system implemented during HIS downtime were beneficial to 80 and 83 users, respectively, highlighting the potential to develop a new HIS downtime response strategy that prioritizes user convenience over reliance on manual processes.

**Table 3:** Results of the user survey for emergency query program and emergency prescription system

Question	*n* (%)
Have you ever experienced a HIS downtime before?	
Yes	43 (46.2)
No	50 (53.8)
Have you ever had to write or carry out a prescription manually?	
Yes	34 (36.6)
No	59 (63.4)
Is your department always prepared for manual prescription writing?	
Prepared	67 (72.0)
Not prepared	26 (28.0)
Is it possible to write or execute a prescription manually during a HIS downtime?	
Always possible	11 (11.8)
Only possible by referring to guidelines	74 (79.6)
Not possible	8 (8.6)
Is the emergency query program helpful during a HIS downtime?	
Helpful	80 (86.0)
Not helpful	13 (14.0)
Is the emergency prescription system helpful during a HIS downtime?	
Helpful	83 (89.2)
Not helpful	10 (10.8)

## Discussion

The widespread adoption of Hospital Information Systems has significantly improved the quality of care. However, as users become more reliant on these systems, their ability to revert to manual processes during HIS downtimes diminishes. This shift calls for the establishment of new response strategies for such disruptions. The advent of low-code platforms has simplified and reduced the costs associated with software development. Notably, Microsoft’s Power Apps enable the rapid creation of customized software solutions that can be conveniently distributed through Microsoft Teams. Ajay et al.‘s research underscores the growing use of low-code platforms in healthcare, exemplified by the creation of a pathology education platform [13]. Our approach harnesses Power Apps and Power Automate to develop software compatible with HIS downtimes, which is then deployed via Microsoft Teams, thus ensuring uninterrupted care.

The feasibility of the new solution for HIS downtime was confirmed through data and user surveys. During the HIS downtime, necessary medications and laboratory tests for patient care were carried out in a balanced manner, and the time taken from the physician’s order to the execution of the prescription by the relevant department was about 18 min, which did not pose a significant obstacle to conducting medical care on site. The user survey provided insights into the existing approach to HIS downtime. Among the respondents, 46% had experienced HIS downtime before, and only 36% had experienced the paper-based process. Notably, only 11% of users reported that the paper-based process was always feasible during HIS downtime. For this reason, 86% and 89% of the respondents respectively indicated that the emergency query program and emergency prescription system were helpful during HIS downtime. This suggests the need for a new strategy to address HIS downtime for users accustomed to modern HIS systems.

However, there are still limitations to our approach in responding to HIS downtime. Although it incurs lower costs compared to traditional development methods, the enterprise-wide adoption of the solution still involves licensing costs, even when using a low-code tool. Additionally, on top of the costs for maintaining the HIS, there are extra costs associated with maintaining and operating a separate solution. Furthermore, most tasks in modern hospitals are performed based on the HIS, and the solution designed for HIS downtime has fundamental limitations in supporting these various services. Lastly, since the new solution is also IT-based, it has a limitation in that it becomes difficult to use in cases where network-level failures occur, rather than issues with the application or database.

Our hospital’s emergency query program and emergency prescription system have shown promise in responding to HIS downtimes, but improvements are needed for stability. The recent HIS downtime was planned for upgrades to the operational server infrastructure, allowing for preparation. However, not all potential HIS downtimes can be anticipated. It is imperative to regularly synchronize the operational server data with the emergency prescription systems to ensure readiness for unplanned outages. Moreover, integrating prescriptions and execution records entered during downtimes into the operational server will prevent data omission. Ongoing user training on process adaptations and emergency systems is crucial to prepare for HIS downtimes. While medical institutions are crafting their own contingency plans, even minimal downtimes for system enhancements are challenging due to the clinical disruptions and potential risks to patient safety. Introducing programs that operate independently of HIS, with user-friendly interfaces, will empower users to better manage during HIS downtimes.

## Conclusions

Essential maintenance-induced HIS downtimes are inherently disruptive to patient care process. Our deployment of an emergency query program and a Microsoft Teams-integrated emergency prescription system demonstrated robust care continuity during HIS downtime.

## Data Availability

The datasets generated and/or analysed during the current study are not publicly available due to the software used during the HIS downtime at our hospital being accessible only within the internal network, and thus, the usage records for it are also accessible only within the internal network of our hospital. However, they are available from the corresponding author on reasonable request.

## Abbreviations

HITECT: Health Information Technology for Economic and Clinical Health
EMR: Electronic medical records
HIS: Hospital information system
DB: Database
OCS: Order communication system
